# Estimating the future UK consultant physician workforce in relation to projected demand on the National Health Service: a modelling study

**DOI:** 10.1016/j.lanepe.2025.101456

**Published:** 2025-09-08

**Authors:** Amar Srinivasa, Kate Lovibond, Emma Carter, Christopher Phillips, Nina Newbery, Nigel Trudgill

**Affiliations:** a Sandwell and West Birmingham NHS Trust, West Bromwich, UK; b Royal College of Physicians, London, UK; c Institute of Cancer and Genomic Sciences, University of Birmingham, UK

**Keywords:** Health policy, Workforce modelling, Workforce planning

## Abstract

**Background:**

The National Health Service (NHS) is facing one of the most challenging periods in its 75-year history. Currently there is a shortage of consultant physicians (senior physicians who have completed specialty training), contributing to a stagnation in productivity alongside increasing demand driven by demographic change. We created a model to predict the future number of NHS consultant physicians, to identify interventions to meet future demand.

**Methods:**

A Markovian approach was used to construct a state transition model, which predicts the number of whole time equivalent (WTE) consultant physicians over the next 25-years starting from 2023. Model states are stages of medical training from medical school to consultant using data from the UK Medical Education Database. Annual transition probabilities were calculated by reviewing the destination of people at each stage of training over consecutive years. With these probabilities, numbers were calculated that progress to a consultant physician post and compared to expected demand predictions.

**Findings:**

The model predicts an approximate 11,000 WTE consultant physician shortfall by 2048 based on high demand predictions. Increasing medical student places to 17,000 per year by 2031, along with increasing retention during physician training through increased training posts, reduced the high demand shortfall to around 5000 WTE. However, increased rates of less than full time working and planned reductions in the intake of overseas doctors would both negatively impact WTE numbers and increase the shortfall from 11,000 to approximately 13,000 and 15,500 respectively by 2048.

**Interpretation:**

Without urgent intervention there will be a substantial shortfall in the number of UK consultant physicians by 2048. Increased medical school places will not meet the demand soon enough or by itself. Increasing training positions will be key to meeting demand, with a continued intake of overseas doctors to stabilise the NHS in the short term.

**Funding:**

The study was commissioned by the 10.13039/501100000395Royal College of Physicians London.

**Research in context**

**Evidence before this study**

There has been limited published research on workforce modelling in the UK to predict if the future healthcare workforce will be able to meet the expected demand. We searched all available resources on the OVID platform for original articles, with no language restrictions using the terms “workforce” AND “modelling” and found 40 publications on predicting future healthcare workforce numbers, primarily modelling single speciality and community medical workforces. The Centre for Workforce Intelligence at the World Health Organisation publish predictions on healthcare workforce for high income countries, with the last report in 2017 suggesting a 33,318 physician surplus in the UK by 2030.

**Added value of this study**

This study is the first to model whole time equivalent (WTE) consultant physician numbers in the UK over a 25 year time period and compare them to demand predictions on a healthcare service. The model was validated using historical data and was 96–98% accurate. This model can be adjusted to assess if healthcare policy around workforce planning will deliver sufficient increase in the workforce to meet expected demand. Additionally, this methodology is transferable to different medical specialities or healthcare systems.

**Implications of all the available evidence**

Data from this study indicate a substantial shortfall of 11,000 WTE consultant physicians by 2048 compared to high demand predictions if there is no change in current policy. The recommendation in the English National Health Service (NHS) Long Term Workforce Plan of increasing medical school places only reduces the shortfall to 9000 WTE. The scenario analyses in this study indicate that multiple interventions are required urgently for the NHS to meet future demands which include: increasing the number of places at medical school; increasing training posts; and continued recruitment of overseas doctors.

## Introduction

2023 marked the 75th anniversary of the NHS and during this period there has been both significant growth and adaptation to meet the changing health needs of the UK population. The NHS currently treats 1.3 million patients a day.^1^ At the heart of the NHS are the staff who deliver this service 21,000 of which in England are consultant physicians.^2^ Consultant physicians, senior physicians who have completed specialty training, significantly contribute to hospital level productivity and are responsible for 29% of outpatient activity.^3^ They play an important role in inpatient flow, ensuring safe admission and discharge from hospital as well as administering care.^4^

Currently the NHS is under extreme pressure and struggling with deteriorating performance against key metrics on treatment and urgent referral waiting times along with heightened bed occupancy, all leading to suboptimal care and decreasing patient satisfaction.^5^, ^6^, ^7^, ^8^ This will likely worsen with a growing elderly population increasing demand for services, as the number of those over 85 years old is projected to double to 3.1 million by 2045.^9^ This will increase admissions associated with multiple comorbidities and complex medical problems, with an increase in average length of inpatient stay and admission rates in the elderly already observed.^10^^,^^11^ In addition, growing levels of dissatisfaction among doctors over pay and working conditions and a lack of training places in the NHS have resulted in significant industrial action among both trainee doctors and consultants in 2023. The General Medical Council's (GMC) National Training Survey reported that a quarter of trainees face high burnout risk, and half of trainers are at moderate to high risk.^12^

It is vital that the NHS plans for the challenging times ahead. A long-term workforce plan for NHS England has been published to address the predicted workforce gap, increase training numbers, improve retention, and reduce current vacancies.^13^ Additionally, workforce plans have been developed for the devolved nations of Scotland, Wales and Northern Ireland.^14^, ^15^, ^16^ Although strategies to accomplish this are welcome, recent figures show that even with increases in doctor numbers, productivity has remained static or worsened. Increased staff churn has contributed to this and the GMC has reported that the number of doctors taking ‘hard steps’ to leave the profession has increased to an all-time high of 7%.^13^^,^^17^ Consequently, 52% of advertised consultant physician posts went unfilled in 2021, 74% due to a lack of any applicant.^18^ This is in contrast to the last report from the World Health Organisation's Centre for Workforce Intelligence, which suggested that there would be a 33,318 physician surplus in the UK by 2030.^19^

We have modelled the numbers of physicians in the UK at various career stages to allow predictions to be made about consultant physician numbers in the future when potential changes are made e.g. to the number of medical school places or increasing training progression through more training positions. The model predictions can then be compared to NHS demand projections from expected demographic changes in the UK population and illustrate if additional interventions are required in planning the future physician workforce.

## Methods

### Model design

A system dynamic modelling approach was used to predict supply of consultant physicians over 25-years starting in 2023. This used a Markov model to simulate movement through different physician training stages to consultant working and incorporated inflows and outflows. The model was built in Excel.

**Fig. 1:**
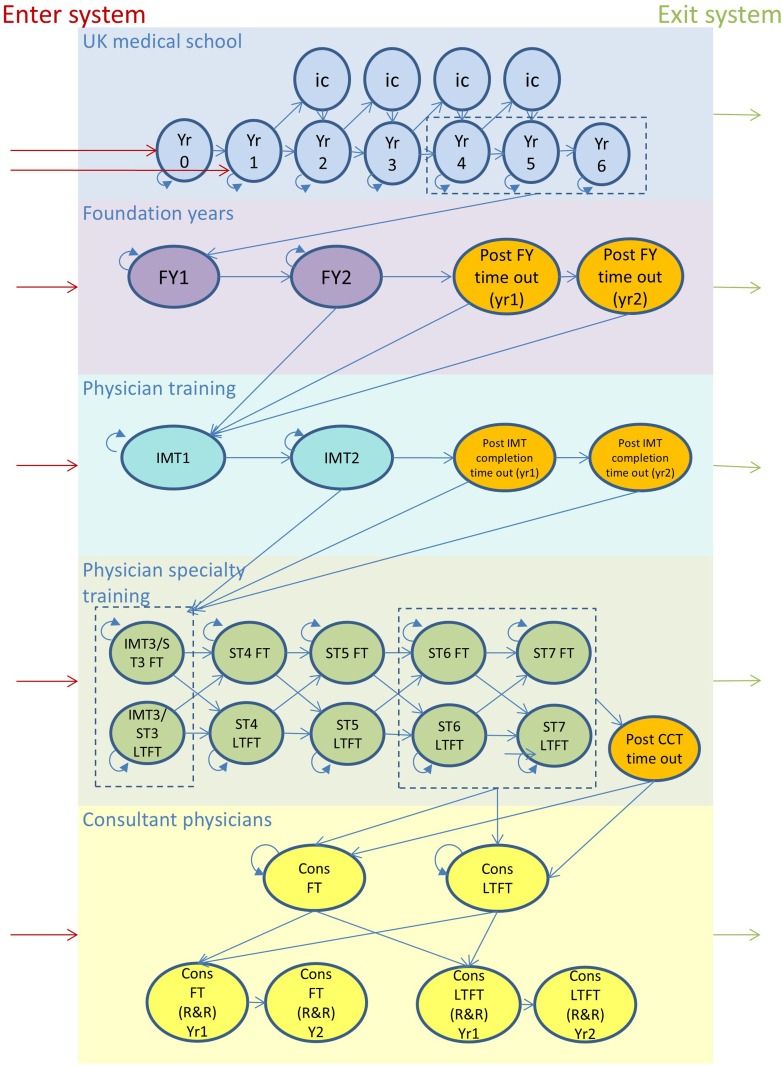
**Model domains and stages on the training pathway for consultant physicians**. Abbreviations: Cons, consultant physician; FY, foundation year; FT, full time; ic, intercalated year; IMT, internal medicine training; LTFT, less than full time; ST, specialist training (physician); R&R retired and returned. Note: There are separate model states by sex. Arrows depict allowed annual transitions. Although not depicted for simplicity, transitions were also allowed forward two ST levels from ST3, ST4 and ST5. Medical school states are based on academic years. Year 6 represents an academic course with mandatory intercalation as this was how it was recorded in the data set. Post-graduate training states are based on training level.

In a Markov model a set of mutually exclusive states are defined along with allowed transitions between states. Model states includes: medical school; foundation years 1 and 2; 2 or 3 years internal medicine training; and 4 or 5 years specialty training (starting at specialty training year 3 following internal medical training year 2 or specialty training year 4 following internal medical training year 3). Separate states were modelled for men and women, less than full time working (LTFT) from Specialty Training onwards, and temporary breaks in training (post completion of foundation years, internal medical training and specialty training). Consultant states also accounted for consultants that retire and return to work. Fig. 1 shows the states and allowed transitions, and inflow and outflow.

Initially, each state was populated with the number of people in 2023. Each cycle (year) people move between states based on defined transition probabilities, allowing the population of each state over time to be simulated. The model allowed for annual external entry (inflow) reflecting: medical school intake; doctors entering training positions from abroad or > 2-year gap in training; and doctors entering consultant positions via non-training routes (Portfolio pathway where a doctor is required to demonstrate equivalent competencies using a portfolio to act as a consultant physician) or from abroad. Annual model exit (outflow) was incorporated, reflecting, for example, leaving the medical profession, non-physician training or over a 2-year training gap.

### Model inputs

Transition and exit probabilities; initial state numbers; and annual model inflows were calculated using the data sources in Supplementary Table S1. For input data see Supplementary Tables S3–S12.

The UK Medical Education Database (UKMED) was the primary source to estimate transition probabilities, initial state numbers and model inflow for medical school and trainee doctors.^20^ UKMED collates data on all UK medical students and all trainee doctors. The extract utilised covered up to 2019/2020 and analyses were conducted using Stata 13. Data from the COVID pandemic years was not utilised because data collection was more limited during this time and was considered likely non-typical in terms of progression and working patterns.

Transition probabilities for undergraduates and trainee doctors were both based on analysis of 2015/2016 and 2016/2017 cohorts, as 3 years follow-up were required to establish break durations. Analyses were restricted to physician specialties (Supplementary Table S2). Annual transition probabilities were calculated by dividing the number making each transition by the total number in the original state (Note: core medical training years 1 and 2 and specialty training year 3 data were used to inform internal medical training year 1 and 2 and internal medical training year 3/specialty training year 3 model states). Those not in training the following year were split into those on a temporary break (in physician training within 3 years) and those exiting physician training (not in physician training within 3 years). Separate analyses were conducted for men and women post-medical school, and LTFT status from specialty training year 3 onwards, as prior to this the rate of progression through training accounted for these factors. Transition probabilities were calibrated to reflect increases in LTFT working and those taking a break post foundation years and internal medical training over time.

UKMED includes numbers who complete training and enter the specialist register. The probability of completing training from UKMED was combined with RCP post-Certificate of Completion of Training (CCT) survey data from 2016 to 2018, to estimate the probability of: completing training and becoming a consultant; completing training and taking a 1-year break before becoming a consultant; and leaving the UK NHS after completing training (exiting the model).^21^ LTFT working was assumed to be the same as in the initial consultant population, regardless of their prior LTFT status.^22^

The probability of exiting the model for any reason (e.g. leaving the NHS or death) from any consultant state was calculated from NHS Electronic Staff Record (ESR) data (England, 2017–2019) from consultants not in a consultant position in the subsequent year. This was used for the whole UK population in the model. The annual retire and return probabilities by sex were estimated using RCP consultant census data, split by LTFT status, assuming that all consultants return for 2 years only.^18^ Exit probabilities for other consultants were calculated from the total exit probabilities, subtracting those who retired and returned.

The latest available cohort from the UKMED dataset (2019/2020) was used to calculate initial numbers in each medical school and trainee doctor state and annual inflows from outside the model and these were adjusted using Office for Students and GMC data to update to 2023 estimates.^23^ From the end of foundation years, inflow was split between those previously in the National Training Survey and those not, as a proxy for those coming from abroad.

Initial numbers for consultant states were based on overall consultant numbers and the proportion by sex and LTFT from the 2023 RCP consultant census, combined with information about retire and return consultants from an analysis of RCP census data 2016–2018.^24^ Annual inflow to consultant states from abroad was estimated using 2020 ESR data uprated to a UK estimate, and to a 2023 estimate based on changes in numbers and LTFT rates over time.^24^ Annual inflow from a non-training route was based on Portfolio pathway/Certificate of Eligibility for Specialist Registration applications granted in physician specialties in the UK in 2023.^25^

### Model outputs

The model predicts whole time equivalent (WTE) consultant physicians over 25 years starting in 2023. LTFT consultants were assumed to work 0.65 WTE on average.^25^

The model was also run for a single year's national intake of medical students to analyse how people moved through training over time and how long it took to become consultant physicians.

Scenario analyses were run altering key model inputs to examine how this affected consultant numbers over time. The basis of some of the modelled interventions comes from the English NHS long term workforce plan such as increasing medical school places and decreasing intake of overseas doctors.^26^ Increases in LTFT working in line with current trends and increased progression through training were also explored.

### Consultant physician demand estimates

Projections of WTE consultant numbers over time were compared to estimated demand that took account of the existing shortfall in consultants and projected increases in demand over time. An initial shortfall of 5.4% was applied based on 2023 NHS England data resulting in an initial WTE demand of 20,935.^27^ Increases in demand over time were from a 2022 Health Foundation report about staff demands over the next 10 years.^28^ The high and medium demand scenarios of a 2.4% and 1.8% annual increase in WTE consultants respectively were applied and assumed to continue over the period of the model.

### Ethics approval

Ethics approval was not required for this study.

### Role of the funding source

The study was commissioned by the Royal College of Physicians London. The funder did not influence the study design, data collection, analysis, interpretation of data, or the writing of the manuscript. The content is the responsibility of the authors and does not necessarily represent the official views of the Royal College of Physicians.

## Results

**Fig. 2:**
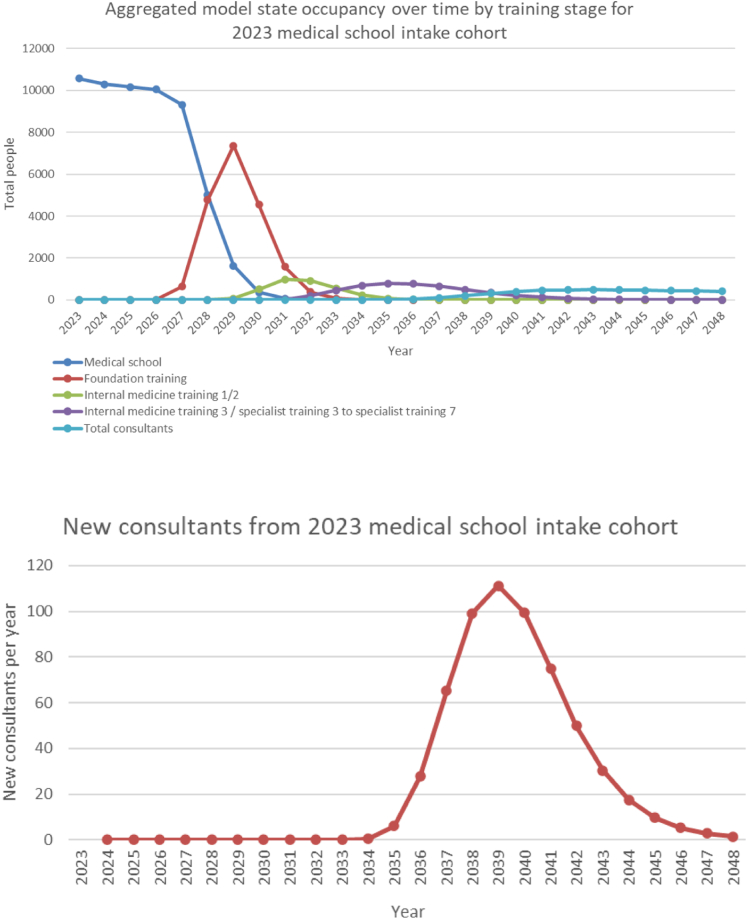
**The progression through the model of a cohort beginning medical school in 2023 and its impact on training grades and consultant physician numbers**.

**Fig. 3:**
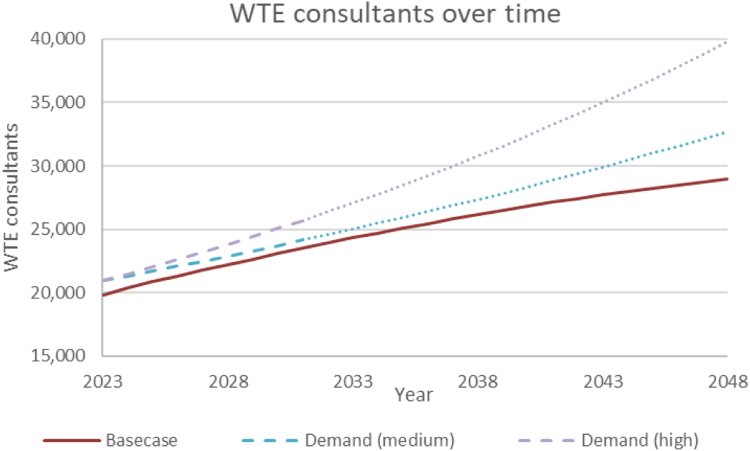
**Modelled whole time equivalent consultant physician numbers compared to estimated demand based on medium (1.8%) and high (2.4%) annual demand increase forecasts**. WTE, Whole time equivalent. Consultant demand estimated based on existing NHS vacancy rates and Heath Foundation estimates of annual demand growth up to 2031 and assumed to continue to increase at the same rate beyond this.

The average time taken to become a consultant was 16.7 years (SD 2.3) (Fig. 2). Consultant physician numbers were predicted to increase over 25 years from approximately 22,000 (2023) to 32,500 (2048). This was estimated to equate to WTE consultants of around 20,000 (2023) to 29,000 (2048). The analysis predicted an approximate 11,000 WTE (27% of the total) and around 4000 WTE (12% of the total) consultant physician shortfall by 2048 compared to high and medium demand predictions, respectively (Fig. 3).

Transition probabilities for physician trainee exit showed that over 30% exited physician training after core medical training (assumed to be same as internal medical training), of these 14% of men and 17% of women changed specialty and 18% and 16% stopped training (defined as not appearing in the NTS dataset for more than 2 years). Across all physician training levels, trainees that exit the model were more likely to stop training than transfer into a non-physician specialty.

### Validation

Using historical data and starting in 2019 the model predicted that the number of consultant physician in 2023 would be 21,302 compared with 22,183 reported by the GMC (96% agreement) and 20,604 compared with 21,102 reported by the GMC (98% agreement) with and without including locums, respectively).^29^

### Scenario analysis

#### Expanding medical school places

**Fig. 4:**
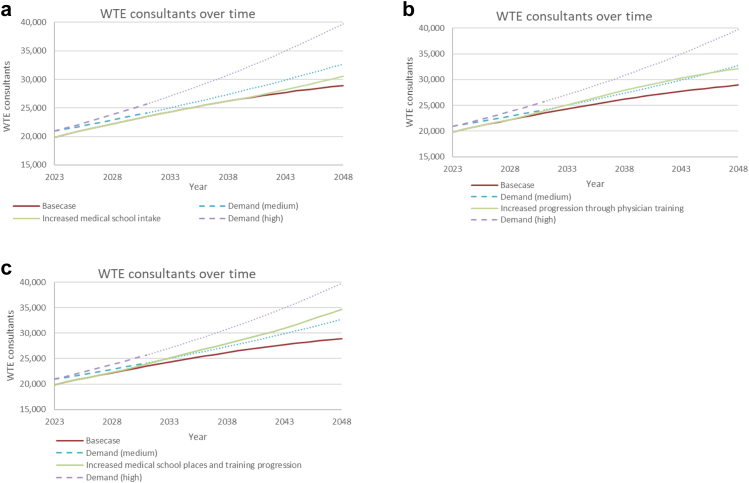
**Positive impact on future whole time equivalent consultant physician numbers and the demand projections for consultant physicians of: (a) Increasing medical school places to 17,000 per annum by 2031; (b) Increased medical trainees progressing into and through physician training at Foundation and Internal Medical Training stages; and (c) The combined effect of increasing medical school places to 17,000 per annum by 2031 and improving progression into and through physician training**. WTE, Whole time equivalent.

The impact of increasing the number of medical school places from around 10,000 to 17,000 per annum by 2031 (assuming linear change over this period) is shown in Fig. 4a. This accounts for the English NHS long term plan aim of increasing places to 15,000 in England and assumes the number from other UK countries remains the same at 2000. New consultant physicians coming from training increased from 1200 to over 1500 in 2048, however, increases only start to appear after 2035. A 9000 WTE and 2000 WTE shortfall remained compared to high and medium demand predictions, respectively.

### Reducing exit from training

Fig. 4b shows the effect of decreasing doctors leaving physician training, which models improved progression through training. The number leaving the specialty and leaving UK medical training after internal medical training were both reduced from 16% to 5%. The number leaving UK medical training after foundation training was reduced from 15% to 5%.

The predicted WTE shortfall in 2048 reduced to 7500 (19% of total demand) and no deficit, compared to high and medium demand predictions, respectively. Most of the impact was from reducing exit after internal medical training. The effects are more rapid than increasing medical school places, beginning 5 years after implementation.

### Combining interventions

The effect of combining increased intake and reduced exit is shown in Fig. 4c. The predicted WTE shortfall reduced to around 5000 (13% of the total) compared to the high demand prediction. The medium demand prediction was met by 2033 and lead to an oversupply of around 2000 WTE consultants by 2048. The number of new consultants from UK medical training per year in 2048 increased from approximately 1200 to over 2000.

### Overseas doctors

**Fig. 5:**
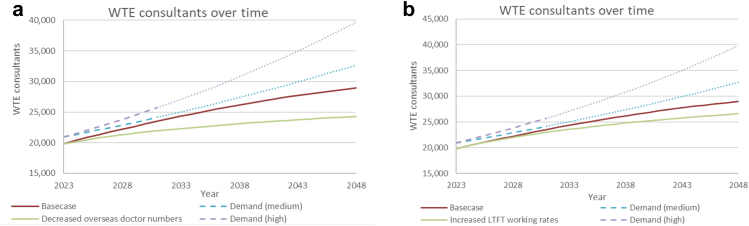
**Negative impact on future whole time equivalent consultant physician numbers and the demand projections for consultant physicians of: (a) Reducing the annual intake of overseas doctors into medical training and consultant posts by 50%. (b) Increasing less than full time working for consultant physicians (by 5%) and specialty trainees (by 1%) per year up to a maximum of 70% for both groups**. WTE, Whole time equivalent.

Fig. 5a shows the effect of halving the inflow from overseas across all levels of training and consultant positions. This results in a consultant physician shortfall of 15,500 WTE (38% of the total) and 6500 WTE by 2048 when compared to the high and medium demand predictions, respectively.

### Less-than full-time working

Fig. 5b illustrates the effect of an increase of 1% per year in the probabilities of people progressing to the next level of specialty training working LTFT combined with an increase of 5% per year in the probabilities of people becoming consultants working LTFT. This increased the 2048 consultant physician shortfall to around 13,000 (33% of total demand) and 6000 WTE compared to high and medium demand predictions, respectively. The resulting LTFT proportions increased from 25% to 54% for specialty training and 31%–50% for consultants and were considered plausible based on extrapolation of historical trends.

## Discussion

At the current trajectory, our model predicts a substantial shortfall by 2048 in the number of WTE consultant physicians of 11,000 WTE and 4000 WTE compared to higher and medium demand prediction levels, respectively. This is likely to be even greater, if the current trend for increasing LTFT working continues. Steps must be taken now to prevent the consultant physician shortfall from increasing and close the gap in staffing. Several potential interventions were modelled: an increase in medical student places to 15,000 per annum in England (17,000 for the UK) by 2032 and improvements to progression in physician training. Increasing medical school places in line with national plans still fails to reach the demand required in 2048 and is contingent on post-graduate training places at all levels also being increased. Increasing progression through physician training was more effective, reaching medium demand projections but with a deficit (19% of total demand) compared to high demand.

Around 800 doctors a year leave UK medical training for over 2 years, which is 7.2% of the physician trainee population. Those that leave UK medical training do so for a variety of reasons, including moving abroad or moving to a non-training post.^30^ Of the 15% that leave after foundation years, this includes those that are unsuccessful in entering specialist training or are undertaking non-training roles. This is one of many bottle necks on the path to becoming a consultant physician, with current a competition ratio at internal medical training of 3.69.^31^ Despite increasing applications, from 2600 in 2015 to 6273 in 2024, the number of internal medical training places has remained static at 1600 places per annum. This bottleneck is then repeated at Specialty Training entry, with virtually all medical specialities having competition ratios over 1, and this increased competition for Specialty Training posts leads to trainees applying to multiple specialties, inflating competition ratios, and as a result some posts are left unfulfilled, as strong candidates may receive multiple offers.^31^^,^^32^ Those who fail to get a training post may explore alternative routes, such as completing their training overseas. In the GMC's Completing the Picture report, of the trainees who desired to return to the NHS, 46.4% would return in a different specialty, with the vast majority returning as a specialist (82.9%), suggesting that a substantial number had completed training overseas.^33^ Another option is non-training posts (locally employed and specialist posts) which have rapidly increased by 40% in last 5 years. However such non-training posts have a high leaver rate, compared to the lower rates in training, suggesting a protective effect of training.^30^ Therefore, increasing progression through training through more training posts will in turn improve retention.

Furthermore, to train medical students and trainee doctors, consultants and general practitioners are needed. As clinical demand is always the priority for frontline clinicians, the capacity to educate medical students and trainees will diminish as clinical demand increases. This is reflected in the results of successive GMC trainer surveys, where over a third of trainers feel they do not have enough time in their job plan to fulfil their role as a trainer.^12^ This area seems to have been overlooked in the planning for a rapid increase in medical school places.

Over recent years, the number of overseas doctors entering the NHS has steadily increased, with 50% of those joining the medical workforce in 2021 being overseas doctors.^34^ Despite their substantial contribution to the NHS, the English NHS Long term workforce plan and more recently in the Ten Year Health Plan for England aims to reduce reliance on overseas staff. In addition, active recruitment from countries, which are already short of doctors, is against the World Health Organisation code of practice for international recruitment.^35^^,^^36^ Reducing the intake of overseas doctors by 50% will increase the consultant physician shortfall by over 60% compared to the medium demand predictions. In addition, unlike the other interventions, overseas doctors entering speciality training have a much more rapid effect on increasing the number of consultant physicians. However, this intervention is unlikely to be a sustainable long-term option, as there are high rates of leaving among overseas doctors.^17^ Alternative options to rapidly address consultant vacancies would include expansion of the Portfolio pathway (formally known as the Certificate of Eligibility for Specialist Registration pathway).

Increasing LTFT working may have profound effects on WTE consultant physician numbers, with an increased shortfall of almost 50% by 2048 compared to medium demand. This is plausible as contractual changes and changing attitudes have made flexible working more readily available. Recent surveys show 50% of consultant physicians would like to work LTFT.^27^ This will delay the effects of any interventions on consultant numbers, as well as total WTEs.^2^^,^^37^ Because of the increase in LTFT working, it is important that future work examines WTE, instead of headcount, which is used in the English NHS long term workforce plan.^26^

Markov modelling is a widely used approach employed in many fields, for example, modelling disease pathways to evaluate healthcare technologies.^38^ It is not a novel approach to workforce planning but there are limited examples in the healthcare sector and none were identified in a UK setting.^39^, ^40^, ^41^ The use of a Markov model approach to predict supply had a number of benefits including that it allowed the time to become a consultant physician to take into account real world data on progression through training that accounts for breaks in training and LTFT working, without having a dataset that followed individuals from starting medical school to becoming a consultant, as transitions in the subsequent year were analysed and used to simulate movement over time. In addition, individual transitions could be modified and the impact on consultant numbers over time evaluated. Evaluation of transition probabilities also allowed insights into the population, for example exit rates at different points in training. This approach was possible due to the availability of the UK Medical Education Database (UKMED), which provided robust, individual-level, national data covering medical school and training allowing calculation of transition probabilities between model states. In contrast, an alternative study modelling the Portuguese physician workforce employed an agent-based simulation approach—more appropriate in contexts where detailed data on individual behaviours, such as movement between public and private practice, are available.^40^^,^^42^

There are, several limitations to our analysis. Transition probabilities were based on analysis of cohorts from 2015/16 and 2016/17. More recent cohorts were not used because we wished to avoid using data from during the COVID pandemic and in order to analyse temporary breaks of up to 2 years, 3 years of follow-up were required. It was considered important to capture breaks in training in order to accurately model the time taken to become a consultant. It also allowed us to estimate the rates of people leaving UK medical training. There are not yet sufficient data post-COVID to re-analyse transition probabilities using this approach. Transition probabilities were calibrated to take account of increased LTFT working since the time of the cohorts up to 2023 and scenario analyses explored potential future increases in LTFT working.

UKMED provided national linked data covering medical school and training informed by routinely collected administrative data. The main limitation was that the dataset only details entry on the specialist register following completion of training, rather than working as a consultant in the UK NHS and so had to be combined with other data sources to estimate transitions to consultant working.

The model reaches 2048, when demand on NHS services will be highest, as people born during the baby boom era (1946–1964) reach approximately 85 years of age. We have used annual demand increases from the Health Foundation, which run until 2031 and extrapolated future demand assuming it will continue to increase at the same rate up to 2048. The Health Foundation projections took account of aging and population health but also the potential for improvements in the productivity of the acute hospital sector due to reductions in the average length of stay and an increasing proportion of elective care delivered through day cases. They were unable to account for other variables that may influence productivity such as advances in healthcare technology, such as artificial intelligence, or future workforce composition (e.g. the contribution of Physician Associates). Beyond 2031 there is additional uncertainty, however, the assumption of continued demand increase was considered reasonable. Demand estimates from a longer-term analysis would be valuable. Additionally there is a drive for more care to be given in the community, but there are similar workforce shortages in community medical specialities such as general practice.^43^ If there is a shortage in general practitioners and limited access to community care, there likely will be more demand on hospitals. Similarly, limitations apply to the supply model, where all inputs were assumed to be constant over time (e.g. exit rates). Consultant physician retirement rates may change as the demographics of the consultant population change and retire and return to work numbers may be overestimated, as not all consultant physician leavers are through retirement. Approximately 25% of the consultant physician workforce will be over 65 in the next 10 years. Given the wealth of experience of senior consultants, further interventions are needed to facilitate consultants to retire and return to work. Other model inputs are necessarily based on historical data, which does not take in to account the effect of the COVID-19 pandemic or trainee doctor and consultants strikes on working or training patterns. In 2012, the unpopular Lansley reforms, had a negative impact on doctors’ numbers, with an increase in the number of consultants leaving the NHS from 5% to 7%.^44^ It is unclear if the COVID pandemic and the current pay disputes will cause a further period of more doctors leaving the NHS. It is also unclear what the effect of the closure of NHS England will have on frontline staff and it is important for the government to provide urgent clarity on addressing workforce shortages. Furthermore, some of the interventions in the recently released Ten Year Health Plan for England, such in increasing training posts are welcomed but the numbers (1000 across all specialities) are unlikely to mitigate other negative impacts such as reduced recruitment of overseas doctors and increased LTFT working and throughout the document there is an assumption that technological advancement will decrease the need for substantial workforce expansion.^36^

In addition, using historical data does not consider the recent change in the structure of physician training in the UK from two years of core medical training to three-years of internal medical training. Exploration of uncertainty focused on varying future model inputs in scenario analyses. Most model inputs are based on novel analysis of real world national datasets and so sensitivity analysis using alternative data sources was not considered appropriate, however, where other similar data analyses were identified, we checked these were consistent or differences could be explained by differences in analysis methods. Future modelling could incorporate probabilistic sensitivity analysis to capture parameter uncertainty in model inputs. Analysis of costs of additional interventions was not part of the scope of this work but is an important topic of future research to guide policy.

### Conclusions

This study suggests that the only way of meeting the future required numbers of WTE consultant physicians will be via a large increase in training places at all stages particularly in internal medical training and speciality training, not simply by increasing medical school places, as suggested in the English NHS long term workforce plan. The model demonstrates the need for overseas doctors to help manage the increase in demand driven by demographic changes and plans to reduce the intake from overseas, although in line with ethical recruitment, will substantially negatively impact the number of WTE consultant physicians when NHS demand will be at its highest. The model also highlights that as more doctors move towards LTFT working, WTE numbers also significantly decrease. The current model can be updated over time to track the predicted numbers of WTE consultant physicians as various workforce policies are implemented. There is limited published research on UK medical workforce modelling and the research team hope that this study will encourage further publications in this important area of public policy, as well as guide future recommendations when the English NHS long term workforce plan it ratified later this year.

## Contributors

Study concept and design was conceived by NT and KL. Data extraction and analysis was performed by KL and EC. KL and EC accessed and verified the data and had full access to the data. Manuscript was drafted by AS. The manuscript was critically reviewed, revised and approved by all authors. All authors were responsible for the decision to submit the manuscript. NT is the senior author for this manuscript.

## Data sharing statement

The data underlying this article were provided by the UK Medical Education Database (UKMED) administered by the General Medical Council, the NHS Electronic Staff Record held by Health Education England and the Medical Workforce Unit of the Royal College of Physicians London and are therefore not available from the authors. A full technical report of the work is available on request.

Source—The UKMED, UKMEDP58 extract was generated on 01/02/2021 and approved for publication by the General Medical Council on [04/12/2024]. We are grateful to UKMED for the use of these data, however, UKMED bears no responsibility for their analysis or interpretation. The data includes information derived from that collected by the Higher Education Statistics Agency Limited (“HESA”) and provided to the GMC (“HESA Data”). Source: HESA Student Records 2002/3 to 2018/19 Copyright Higher Education Statistics Agency Limited. The Higher Education Statistics Agency Limited makes no warranty as to the accuracy of the HESA Data and cannot accept responsibility for any inferences or conclusions derived by third parties from data or other information supplied by it.

## Declaration of interests

No conflicts of interest to declare.
