# Supplementary material for: Estimating the future UK consultant physician workforce in relation to projected demand on the National Health Service: a modelling study
Source: Lancet Reg Health Eur. 2025 Sep 8;58:101456. doi: 10.1016/j.lanepe.2025.101456 (PMC12451377; doi:10.1016/j.lanepe.2025.101456)
Supplement: Supplementary Material [file mmc1.docx]

**Supplementary material**

Tables that include data from the analysis of the UKMED dataset (either directly or combined with other data) are reported in line with UKMED Statistical Disclosure controls. Numbers are rounded to the nearest multiples of 5 and percentages based on fewer than 22.5 individuals are suppressed (*). Numbers may therefore not add up to totals shown. Calculations were based on the original data not the rounded numbers shown.

**Data sources**

Model inputs are based on national UK datasets for medical students, post-graduate trainees and consultants where available. Where data from NHS England was used this was either assumed to be representative of the UK (e.g. probability of exit from consultant states) or uprated to a UK estimate (e.g. annual consultant inflow from abroad). Data sources are summarised in table 1.

Initial population numbers are estimates for 2023. Note that the model was initially developed with a start year of 2019 as this was the generally the latest available data at the time and so data analyses refer to this. However, the model was updated to have a start year of 2023 by adjusting initial population numbers for each state to reflect published 2023 data. Inflow was also adjusted to reflect 2023 estimates. Transition probabilities were calibrated to reflect 2023 LTFT working rates. Specifics of how data sources were combined for model inputs are given under the model input data tables below. More detailed information is available in a technical report available on request.

**Supplementary Table** **1:** Data sources for the model

| States | Initial numbers (2023) | Transition and exit probabilities | Annual model inflow |
| --- | --- | --- | --- |
| Medical training | - UKMED (HESA) analysis (2019/2020 cohort) [1] - Uprated to 2023 estimates using Office for Students UK medical school intake data (2020 to 2023) combined with model transition probabilities [2] | - UKMED (HESA/NTS) analysis (2015/16 and 2016/17 cohorts) [1] | - 2023 Office for Students UK medical school intake data for initial numbers [2] - UKMED (HESA) analysis (2019/2020 cohort) (foundation year) [1] |
| Post-graduate training and breaks | - UKMED (NTS) analysis (2019/2020 cohort) [1] - Uprated to 2023 estimates using GMC reported increases in trainee numbers and LTFT working (2019 to 2023) [3, 4] | - UKMED (NTS) analysis (2015/16 and 2016/17 cohorts) [1] - RCP post-CCT survey (2016-2018**) [5]   Adjustments to account for increases in LTFT working over time uses GMC reported NTS LTFT data over time (to 2023) and 2023 RCP consultant census for initial numbers | - UKMED (NTS) analysis (2019/2020 cohort) [1] - Uprated to 2023 estimates using GMC reported trainee numbers over time (2019 to 2023) |
| Consultants | - 2023 RCP consultant census [6] - Retire and return estimated using 2016-2018* data [7] | - Retire and return informed by 2016-2018* RCP consultant census - Exit informed by NHS England ESR data (2017-2019) [7] | - 2023 GMC specialist register data (inflow via portfolio pathway) [8] - NHS England ESR 2020 data (inflow from abroad); uprated to UK estimate [7] - Uprated to 2023 estimate using RCP consultant census data |

*2019 and 2020 data not used as higher rates in these years considered a temporary effect due to pension changes that incentivised retiring and returning.

**2019/2020 combined data was not used to avoid using data that might be impacted by the COVID pandemic years.

CCT- Completion of Training Certificate

ESR- Electronic Staff Record

GMC- General Medical Council

HESA- Higher Education Statistics Agency

LTFT- Less than full time

NHS- National Health Service

NTS – National Training Survey

RCP - The Royal College of Physicians

UKMED- The UK Medical Education Database

**Supplementary Table 2**

**UKMED specialities included in the model data**

In the analysis of UKMED NTS data doctors were categorised as in physician training on the following basis.

| **Training_level_2018** | **Programme_speciality_2018** |
| --- | --- |
| F1-2 | - Foundation programme |
| CT1-3 | - ~~Acute care common stem~~ |
|  | - ~~Broad based training~~ |
|  | - ~~Core anaesthetics training~~ |
|  | - Core medical training |
|  | - ~~Core psychiatry training~~ |
|  | - ~~Core surgical training~~ |
| ST1-8 | 1. Acute internal medicine |
|  | 1. Allergy |
|  | 1. ~~Anaesthetics~~ |
|  | 1. Audio vestibular medicine |
|  | 1. Aviation and space medicine |
|  | 1. ~~Cardio-thoracic surgery~~ |
|  | 1. Cardiology |
|  | 1. ~~Chemical pathology~~ |
|  | 1. ~~Child and adolescent psychiatry~~ |
|  | 1. Clinical genetics |
|  | 1. Clinical neurophysiology |
|  | 1. ~~Clinical oncology~~ |
|  | 1. Clinical pharmacology and therapeutics |
|  | 1. ~~Clinical radiology~~ |
|  | 1. Combined infection training |
|  | 1. Community sexual and reproductive health |
|  | 1. Dermatology |
|  | 1. ~~Diagnostic neuropathology~~ |
|  | 1. ~~Emergency medicine~~ |
|  | 1. Endocrinology and diabetes mellitus |
|  | 1. ~~Forensic histopathology~~ |
|  | 1. ~~Forensic psychiatry~~ |
|  | 1. Gastroenterology |
|  | 1. General (internal) medicine |
|  | 1. ~~General practice~~ |
|  | 1. ~~General surgery~~ |
|  | 1. ~~General psychiatry~~ |
|  | 1. Genito-urinary medicine |
|  | 1. Geriatric medicine |
|  | 1. Haematology |
|  | 1. ~~Histopathology~~ |
|  | 1. Immunology |
|  | 1. Infectious diseases |
|  | 1. ~~Intensive care medicine~~ |
|  | 1. ~~Intensive care medicine – prehospital~~ |
|  | 1. ~~Intensive care medicine – single~~ |
|  | 1. ~~Medical virology~~ |
|  | 1. ~~Medical microbiology~~ |
|  | 1. Medical oncology |
|  | 1. Medical ophthalmology |
|  | 1. ~~Medical psychotherapy~~ |
|  | 1. Neurology |
|  | 1. ~~Neurosurgery~~ |
|  | 1. Nuclear medicine |
|  | 1. ~~Obstetrics and gynaecology~~ |
|  | 1. ~~Occupational medicine~~ |
|  | 1. ~~Old age psychiatry~~ |
|  | 1. ~~Ophthalmology~~ |
|  | 1. ~~Oral and maxillo-facial surgery~~ |
|  | 1. ~~Otolaryngology~~ |
|  | 1. ~~Paediatric and perinatal pathology~~ |
|  | 1. Paediatric cardiology |
|  | 1. ~~Paediatric surgery~~ |
|  | 1. ~~Paediatrics~~ |
|  | 1. Palliative medicine |
|  | 1. Pharmaceutical medicine |
|  | 1. ~~Plastic surgery~~ |
|  | 1. ~~Psychiatry of learning disabilities~~ |
|  |  |
|  | 1. Rehabilitation medicine |
|  | 1. Renal medicine |
|  | 1. Respiratory medicine |
|  | 1. Rheumatology |
|  | 1. Sport and exercise medicine |
|  | 1. ~~Trauma and orthopaedic surgery~~ |
|  | 1. ~~Urology~~ |
|  | 1. ~~Vascular surgery~~ |

**Initial population numbers**

The model was initially populated with estimates of people in each state in 2023 as described below in tables 3 to 5.

**Supplementary Table 3: Initial population number by model state: medical school states**

| **Model state** | **Female** | | **Male** | |
| --- | --- | --- | --- | --- |
| Year 0 / Foundation or pre-entry year (intake) | 320 | 67% | 160 | 33% |
| Year 1 (intake and existing) | 6600 | 62% | 3975 | 38% |
| Intercalating after year 1 | 10 | * | 5 | * |
| Year 2 | 6205 | 62% | 3735 | 38% |
| Intercalating after year 2 | 665 | 62% | 400 | 38% |
| Year 3 | 6935 | 62% | 4180 | 38% |
| Intercalating after year 3 | 1130 | 62% | 685 | 38% |
| Year 4 | 6270 | 62% | 3825 | 38% |
| Intercalating after year 4 | 515 | 61% | 330 | 39% |
| Year 5 | 4970 | 61% | 3225 | 39% |
| Year 6 | 435 | 59% | 305 | 41% |
| Sub totals | 33,735 | 62% | 20,665 | 38% |
| TOTAL | 54,400 | | | |

*Source: Estimated using model transition probabilities, 2019 initial numbers from analysis of UKMED, HESA 2019 data (2017 for intercalation states) and national year 1 medical school national intake data 2020 to 2023.* *Numbers are rounded to the nearest multiples of 5 and percentages based on fewer than 22.5 individuals are suppressed (*).*

*Note: Most people will have 5 years at medical school with an intercalation year optional. People on a graduate programme will have 4 with an intercalation year optional. A small number of medical school courses include a mandatory intercalation year and this is sometimes recorded in the data as an additional year of medical school (rather than an intercalation year) and so the course length is 6 years. Year 0 is for courses that include a foundation year.*

**Supplementary Table 4: Initial population number by model state: post-graduate physician training**

| **Training level** | **Model state** | **Female** | | **Male** | | **Total** |
| --- | --- | --- | --- | --- | --- | --- |
| Foundation | F1 | 4490 | 54% | 3,765 | 46% | 17,165 |
|  | F2 (in training only) | 4370 | 55% | 3,535 | 45% |  |
|  | Post F2 break (yr 1) | 440 | 55% | 355 | 45% |  |
|  | Post F2 break (yr 2) | 115 | 54% | 95 | 46% |  |
| IMT1 to 2 | IMT1 | 990 | 54% | 855 | 46% | 4,195 |
|  | IMT2 (in training only) | 895 | 55% | 735 | 45% |  |
|  | Post IMT completion break (yr 1) | 315 | 54% | 275 | 46% |  |
|  | Post IMT completion break (yr 2) | 70 | 54% | 60 | 46% |  |
| I IMT3/ST3 to ST7 | IMT3/ST3 FT | 640 | 45% | 790 | 55% | 17,165 |
|  | ST4 FT | 525 | 44% | 670 | 56% |  |
|  | ST5 FT | 465 | 41% | 675 | 59% |  |
|  | ST6 FT | 320 | 34% | 615 | 66% |  |
|  | ST7 FT | 215 | 33% | 440 | 67% |  |
|  | IMT3/ST3 LTFT | 295 | 84% | 55 | 16% |  |
|  | ST4 LTFT | 280 | 92% | 25 | 8% |  |
|  | ST5 LTFT | 390 | 90% | 45 | 10% |  |
|  | ST6 LTFT | 440 | 93% | 35 | 7% |  |
|  | ST7 LTFT | 220 | 86% | 35 | 14% |  |
| Post-CCT break |  | 45 | 51% | 40 | 49% |  |

*Source: Analysis of UKMED 2019 NTS data uprated using increases in numbers and LTFT working 2019 to 2023 from the GMC data explorer online. Numbers are rounded to the nearest multiples of 5 and percentages based on fewer than 22.5 individuals are suppressed (*).*

*Abbreviations: CCT = certificate of completion of training; FT = full-time; IMT = internal medicine training; LTFT = less-than full-time; ST = specialist training.*

*Note: people were considered to be on a temporary break if they returned to physician training after 1 or 2 years; people who had a longer break were considered to have left physician training in the model. Training state numbers are people in training and on temporary breaks in training unless specified.*

**Supplementary Table 5: Initial population number by model state: consultant states**

| **Model state** | **Female** | | **Male** | |
| --- | --- | --- | --- | --- |
| Consultant not R&R FT | 4874 | 32% | 10,226 | 68% |
| Consultant not R&R LTFT | 3984 | 66% | 2,089 | 34% |
| Consultant R&R FT Yr1 | 15 | 15% | 83 | 85% |
| Consultant R&R FT Yr2 | 15 | 15% | 83 | 85% |
| Consultant R&R LTFT Yr1 | 80 | 19% | 345 | 81% |
| Consultant R&R LTFT Yr2 | 80 | 19% | 345 | 81% |
| Subtotals | 9048 | 41% | 13,171 | 59% |
| TOTAL | 22,219 | | | |

*Source: Estimated using RCP consultant census data (2023 for overall numbers, % female and FT/LTFT by female/male; R&R 2016-2018)*

*Abbreviations: FT = full-time; LTFT = less-than full-time; R&R = retired and returned to work; Yr = year*

**Transition probabilities**

Each year moves between model states as defined by the transition probabilities in Supplementary Tables 5 to 8.

**Supplementary Table 6: Transition probabilities: medical school states**

| **Destination** | **Medical school state** | | | | | | | | | | |
| --- | --- | --- | --- | --- | --- | --- | --- | --- | --- | --- | --- |
|  | **0** | **1** | **IC1** | **2** | **IC2** | **3** | **IC3** | **4** | **IC4** | **5** | **6** |
| Progress to next academic year | 71% | 92% | 44% | 86% | 99% | 73% | 98% | 75% | 98% | 9% |  |
| Repeat the year | 5% | 5% |  | 3% |  | 9% |  | 5% |  | 5% | 2% |
| Intercalate |  | 0.1% |  | 10% |  | 17% |  | 9% |  |  |  |
| Exit - Stop training | 24% | 3% | 56% | 1% | 1% | 1% | 2% | 0.6% | 2% | 0.6% | 0.0% |
| Exit - graduate but leave medicine |  |  |  |  |  |  |  | 0.3% |  | 1.5% | 2.8% |
| Graduate and progress to FY1 (yr4-6) |  |  |  |  |  |  |  | 10% |  | 83% | 95% |

*Source: Analysis of UKMed, HESA 2015/2016 and 2016/2017 cohorts.*

*Abbreviations: ICX = intercalating after year X; FY = foundation training year 1.*

*Note: Most people will have 5 years at medical school with an intercalation year optional. People on a graduate programme will have 4 with an intercalation year optional. A small number of medical school courses include a mandatory intercalation year, and this is sometimes recorded in the data as an additional year of medical school (rather than an intercalation year) and so the course length is 6 years. Year 0 is foundation year.*

**Supplementary Table 7:Transition probabilities: post-graduate medical training states (female)**

|  | **FY1** | **FY2** | **Time out FY2 Yr1** | **Time out FY2 Yr2** | **IMT1** | **IMT2** | **Time out (IMT2 >ST3) Yr1** | **Time out (IMT2 >ST3) Yr2** | **IMT3/ ST3 (FT)** | **ST4 (FT)** | **ST5 (FT)** | **ST6 (FT)** | **ST7 (FT)** | **IMT3/ ST3 (LTFT)** | **ST4 (LTFT)** | **ST5 (LTFT)** | **ST6 (LTFT)** | **ST7 (LTFT)** | **Time out (post CCT)** |
| --- | --- | --- | --- | --- | --- | --- | --- | --- | --- | --- | --- | --- | --- | --- | --- | --- | --- | --- | --- |
| Progress 1 level (FT/LTFT) | 95% | 10% | 72% | 100% | 88% | 37% | 70% | 100% | 69% | 68% | 58% | 43% |  | 42% | 40% | 41% | 23% |  |  |
| % of those that progress progress |  |  |  |  |  | 56% |  |  | 23% | 38% | 38% | 25% |  | 80% | 81% | 81% | 78% |  |  |
| Progress 1 level FT |  |  |  |  |  | 16% |  |  | 53% | 42% | 36% | 32% |  | 8% | 7% | 8% | 5% |  |  |
| Progress 1 level LTFT |  |  |  |  |  | 21% |  |  | 16% | 26% | 22% | 11% |  | 33% | 32% | 33% | 18% |  |  |
| Progress 2 levels FT |  |  |  |  |  |  |  |  | 6% | 5% | 5% |  |  |  |  |  |  |  |  |
| Progress 2 levels LTFT |  |  |  |  |  |  |  |  |  |  |  |  |  | 8% | 6% | 3% |  |  |  |
| Stay at level | 2% | 1% |  |  | 5% | 6% |  |  | 20% | 18% | 26% | 19% | 10% | 42% | 45% | 42% | 43% | 30% |  |
| Exit model – non-physician training | 0.3% | 68% |  |  | 5% | 18% |  |  | 1.5% | 1.1% | 0.3% | 0.1% | 0.0% | 2.0% | 1.8% | 1.2% | 0.7% | 1.0% |  |
| Exit model - stop training | 2.4% | 13% |  |  | 2% | 15% |  |  | 4% | 8% | 12% | 5% | 2% | 7% | 8% | 13% | 1% | 1% |  |
| Temp time out |  | 7% |  |  |  | 25% |  |  |  |  |  |  |  |  |  |  |  |  |  |
| Time out y2 |  |  | 28% |  |  |  | 30% |  |  |  |  |  |  |  |  |  |  |  |  |
| Complete training and join SR |  |  |  |  |  |  |  |  |  |  |  | 34% | 88% |  |  |  | 33% | 68% |  |
| Consultant FT |  |  |  |  |  |  |  |  |  |  |  | 16% | 42% |  |  |  | 16% | 32% | 55% |
| Consultant LTFT |  |  |  |  |  |  |  |  |  |  |  | 13% | 34% |  |  |  | 13% | 26% | 45% |
| Temp time out post-CCT |  |  |  |  |  |  |  |  |  |  |  | 2% | 6% |  |  |  | 2% | 5% |  |
| Exit model post ST |  |  |  |  |  |  |  |  |  |  |  | 2% | 6% |  |  |  | 2% | 5% |  |
| Exit total | 3% | 82% |  |  | 7% | 33% |  |  | 6% | 9% | 12% | 7% | 8% | 9% | 10% | 14% | 4% | 7% |  |

*Sources: Analysis of UKMed, NTS 2015/2016 cohorts and RCP post-CCT survey data 2016-2018; LTFT adjusted using 2023 NTS data from GMC reporting tool and 2023 RCP consultant census. Abbreviations: FY = foundation training year, IMT – Internal Medical Trainee, ST= Speciality Trainee, CCT= Certificate of completion of Training*

*Note: numbers in grey are totals or calculations using other data in table.*

**Supplementary Table 8: Transition probabilities: post-graduate medical training states (male)**

|  | **FY1** | **FY2** | **Time out FY2 Yr1** | **Time out FY2 Yr2** | **IMT1** | **IMT2** | **Time out (IMT2 >ST3) Yr1** | **Time out (IMT2 >ST3) Yr2** | **IMT3/ ST3 (FT)** | **ST4 (FT)** | **ST5 (FT)** | **ST6 (FT)** | **ST7 (FT)** | **IMT3/ ST3 (LTFT)** | **ST4 (LTFT)** | **ST5 (LTFT)** | **ST6 (LTFT)** | **ST7 (LTFT)** | **Time out (post CCT)** |
| --- | --- | --- | --- | --- | --- | --- | --- | --- | --- | --- | --- | --- | --- | --- | --- | --- | --- | --- | --- |
| Progress 1 level (FT/LTFT) | 96% | 11% | 67% | 100% | 92% | 43% | 71% | 100% | 73% | 73% | 62% | 50% |  | 74% | 48% | 54% | 34% |  |  |
| % LTFT |  |  |  |  |  | 8% |  |  | 7% | 10% | 6% | 10% |  | 38% | 86% | 39% | 29% |  |  |
| Progress 1 level FT |  |  |  |  |  | 40% |  |  | 68% | 66% | 59% | 45% |  | 46% | 7% | 33% | 24% |  |  |
| Progress 1 level LTFT |  |  |  |  |  | 3% |  |  | 5% | 7% | 3% | 5% |  | 28% | 41% | 21% | 10% |  |  |
| Progress 2 levels FT |  |  |  |  |  |  |  |  | 6% | 6% | 6% |  |  |  |  |  |  |  |  |
| Progress 2 levels LTFT |  |  |  |  |  |  |  |  |  |  |  |  |  | 11% | 12% | 10% |  |  |  |
| Stay at level | 1% | 1% |  |  | 1% | 3% |  |  | 15% | 11% | 16% | 16% | 12% | 13% | 31% | 22% | 29% | 27% |  |
| Exit model - non-physician training | 0.4% | 64% |  |  | 4% | 14% |  |  | 1.2% | 1.4% | 0.7% | 0.5% | 0.3% | 0.0% | 1.5% | 2.9% | 4.3% | 0.0% |  |
| Exit model - stop training | 2.2% | 18% |  |  | 3% | 18% |  |  | 4% | 8% | 16% | 3% | 2% | 3% | 7% | 12% | 6% | 2% |  |
| Temp time out |  | 7% |  |  |  | 22% |  |  |  |  |  |  |  |  |  |  |  |  |  |
| Time out y2 |  |  | 33% |  |  |  | 29% |  |  |  |  |  |  |  |  |  |  |  |  |
| Complete training and join SR |  |  |  |  |  |  |  |  |  |  |  | 30% | 86% |  |  |  | 27% | 70% |  |
| Consultant FT |  |  |  |  |  |  |  |  |  |  |  | 21% | 61% |  |  |  | 19% | 50% | 83% |
| Consultant LTFT |  |  |  |  |  |  |  |  |  |  |  | 4% | 13% |  |  |  | 4% | 10% | 17% |
| Temp time out post-CCT |  |  |  |  |  |  |  |  |  |  |  | 2% | 6% |  |  |  | 2% | 5% |  |
| Exit model post ST |  |  |  |  |  |  |  |  |  |  |  | 2% | 6% |  |  |  | 2% | 5% |  |
| Exit total | 3% | 81% |  |  | 7% | 32% |  |  | 6% | 10% | 17% | 5% | 8% | 3% | 9% | 14% | 12% | 7% |  |

*Source: Analysis of UKMed, NTS 2015/2016 cohorts and RCP post-CCT survey data; LTFT adjusted using 2023 NTS data from GMC reporting tool and 2023 RCP consultant census. Abbreviations: FY = foundation training year. , IMT – Internal Medical Trainee, ST= Speciality Trainee, CCT= Certificate of completion of Training*

*Note: numbers in grey are totals or calculations using other data in table.*

Supplementary Table 9: Transition probabilities: consultant states

| Destination | Consultant states | | | | | |
| --- | --- | --- | --- | --- | --- | --- |
|  | Cons (FT) | Cons (LTFT) | Cons R&R (FT) Yr1 | Cons R&R (LTFT) Yr1 | Cons R&R (FT) Yr2 | Cons R&R (LTFT) Yr2 |
| **Female** | | | | | | |
| Cons (FT) | 95% |  |  |  |  |  |
| Cons (LTFT) |  | 95% |  |  |  |  |
| Cons R&R (FT) Yr1 | 0.2% | 0.2% |  |  |  |  |
| Cons R&R (LTFT) Yr1 | 0.9% | 0.9% |  |  |  |  |
| Cons R&R (FT) Yr2 |  |  | 100% |  |  |  |
| Cons R&R (LTFT) Yr2 |  |  |  | 100% |  |  |
| Exit model | 3.5% | 3.5% | 0% | 0% | 100% | 100% |
| **Male** | | | | | | |
| Cons (FT) | 95% |  |  |  |  |  |
| Cons (LTFT) |  | 95% |  |  |  |  |
| Cons R&R (FT) Yr1 | 0.7% | 0.7% |  |  |  |  |
| Cons R&R (LTFT) Yr1 | 2.8% | 2.8% |  |  |  |  |
| Cons R&R (FT) Yr2 |  |  | 100% |  |  |  |
| Cons R&R (LTFT) Yr2 |  |  |  | 100% |  |  |
| Exit model | 1.6% | 1.6% | 0% | 0% | 100% | 100% |

*Source: estimated using HEE 2017-2019 ESR data and 2016-2018 RCP consultant census data.*

*Abbreviations: Cons = consultant; FT = full-time; LTFT = less-than full-time; R&R = retired and returned to work; Yr = year.*

**Annual model inflow**

Each year people entered the model as shown in Supplementary Tables 9 to 11.

**Supplementary Table 10: Annual model inflow: medical school intake**

| Model state | Female | | Male | |
| --- | --- | --- | --- | --- |
| Year 0 / Foundation or pre-entry year | 305 | 66% | 155 | 34% |
| Year 1 | 6030 | 62% | 3655 | 38% |
| Sub totals | 6340 | 62% | 3810 | 38% |
| TOTAL | 10,150 | | | |

*Source: 2023 UK medical school intake Office for Students; adjusted using foundation year estimates from analysis of UKMED, HESA 2019/2020 data. Numbers are rounded to the nearest multiples of 5 and percentages based on fewer than 22.5 individuals are suppressed (*).*

**Supplementary Table 11: Annual model inflow: post-graduate training states from outside the model**

| **Model state** | **Female** | | **Male** | | **Total** |
| --- | --- | --- | --- | --- | --- |
| **Inflow from abroad** | | | | | |
| F1 | 245 | 52% | 225 | 48% | 465 |
| F2 | 85 | 47% | 95 | 53% | 175 |
| IMT1 | 135 | 41% | 195 | 59% | 330 |
| IMT2 | None applied | | | | |
| IMT3/ST3 FT | 90 | 36% | 155 | 64% | 245 |
| ST4 FT | 5 | * | 10 | * | 15 |
| ST5 FT | None applied | | | | |
| ST6 FT | None applied | | | | |
| ST7 FT | None applied | | | | |
| IMT3/ST3 LTFT | 5 | * | 0 | * | 5 |
| ST4 LTFT | None applied | | | | |
| ST5 LTFT | None applied | | | | |
| ST6 LTFT | None applied | | | | |
| ST7 LTFT | None applied | | | | |
| **Other inflow (from non-physician medical training or from break in NTS of >2 years)** | | | | | |
| F1 | None applied | | | | |
| F2 | None applied | | | | |
| IMT1 | 25 | 52% | 20 | 48% | 45 |
| IMT2 | None applied | | | | |
| IMT3/ST3 FT | 50 | 43% | 65 | 57% | 120 |
| ST4 FT | 15 | * | 10 | * | 20 |
| ST5 FT | 20 | 34% | 40 | 66% | 60 |
| ST6 FT | 30 | 39% | 50 | 61% | 85 |
| ST7 FT | 20 | 37% | 35 | 63% | 55 |
| IMT3/ST3 LTFT | 10 | * | 0 | * | 15 |
| ST4 LTFT | 5 | * | 0 | * | 5 |
| ST5 LTFT | 10 | * | 0 | * | 15 |
| ST6 LTFT | 15 | * | 0 | * | 15 |
| ST7 LTFT | 5 | * | 0 | * | 10 |

*Source: Analysis of UKMED, NTS 2019/2020 data; uprated to 2023 estimates using change over time 2019-2023. Numbers are rounded to the nearest multiples of 5 and percentages based on fewer than 22.5 individuals are suppressed (*).*

*Abbreviations: F = foundation year; FT = full-time; IMT = internal medicine training; LTFT = less-than full-time; ST = specialty training.*

**Supplementary Table 12: Annual model inflow: consultant states from outside the model**

| **Model state** | **Female** | | **Male** | | **Total** |
| --- | --- | --- | --- | --- | --- |
| **Inflow from abroad** | | | | | |
| Consultant (not R&R) FT | 80 | 26% | 223 | 74% | 303 |
| Consultant (not R&R) LTFT | 65 | 59% | 46 | 41% | 111 |
| **Other inflow (CESR route)** | | | | | |
| Consultant (not R&R) FT | 31 | 33% | 64 | 67% | 95 |
| Consultant (not R&R) LTFT | 25 | 66% | 13 | 34% | 38 |

*Abbreviations: CESR = certificate of eligibility for specialist registration; FT = full-time; LTFT = less-than full-time; R&R = retired and returned to work.*

**References**

1. *GMC. UKMEDP58. UK Medical Education Database [dataset]. Extract last generated 01/02/2021.* [*https://www.ukmed.ac.uk/*](https://www.ukmed.ac.uk/).

2. *Office for Students. Medical and dental students survey 2023 intake results for 2022-23 and 2023-24 academic years [online]. 2023.* [*https://www.officeforstudents.org.uk/advice-and-guidance/funding-for-providers/health-education-funding/medical-and-dental-intakes/*](https://www.officeforstudents.org.uk/advice-and-guidance/funding-for-providers/health-education-funding/medical-and-dental-intakes/) *(accessed 29 January 2024)*.

3. *GMC. GMC data explorer: post-graduate trainees summary data [online].* [*https://gde.gmc-uk.org/postgraduate-training/postgraduate-trainees/postgraduate-trainees-summary-data*](https://gde.gmc-uk.org/postgraduate-training/postgraduate-trainees/postgraduate-trainees-summary-data) *(accessed 29 January 2024)*.

4. *GMC. GMC Education data tool: Other NTS reports, Less than full time [online].* [*https://edt.gmc-uk.org/other-nts-reports/less-than-full-time-ltft*](https://edt.gmc-uk.org/other-nts-reports/less-than-full-time-ltft) *(accessed 20 March 2024).*

5. *Personal communication. Medical Workforce Unit, Royal College of Physicians. Post-CCT survey 2009-2020.* .

6. *Personal communication. Medical Workforce Unit, Royal College of Physicians. Consultant census 2023.*

7. *Personal communication. Health education England. NHS employee service record. Source and destination of consultant physicians year on year 2014-2020.* .

8. *GMC. Certificates of Eligibility on to the Specialist Register (CESR) completed 2023 [online]. cesr-cegprs-granted-q4-2023_pdf-105165276.pdf (gmc-uk.org) (accessed 4 March 2024)*
